# Turning a Collagenesis-Inducing Peptide Into a Potent Antibacterial and Antibiofilm Agent Against Multidrug-Resistant Gram-Negative Bacteria

**DOI:** 10.3389/fmicb.2019.01915

**Published:** 2019-08-20

**Authors:** Ana Gomes, Lucinda J. Bessa, Iva Fernandes, Ricardo Ferraz, Nuno Mateus, Paula Gameiro, Cátia Teixeira, Paula Gomes

**Affiliations:** ^1^Laboratório Associado para a Química Verde da Rede de Química e Tecnologia (LAQV-REQUIMTE), Departamento de Química e Bioquímica, Faculdade de Ciências, Universidade do Porto, Porto, Portugal; ^2^Ciências Químicas e das Biomoléculas, Escola Superior de Saúde, Politécnico do Porto, Porto, Portugal

**Keywords:** antibiofilm, antimicrobial peptide, collagen, ESKAPE, *Klebsiella pneumoniae*, multidrug-resistant bacteria, wound-healing

## Abstract

Antimicrobial resistance is becoming one the most serious health threats worldwide, as it not only hampers effective treatment of infectious diseases using current antibiotics, but also increases the risks of medical procedures like surgery, transplantation, bone and dental implantation, chemotherapy, or chronic wound management. To date, there are no effective measures to tackle life-threatening nosocomial infections caused by multidrug resistant bacterial species, of which Gram-negative species within the so-called “ESKAPE” pathogens are the most worrisome. Many such bacteria are frequently isolated from severely infected skin lesions such as diabetic foot ulcers (DFU). In this connection, we are pursuing new peptide constructs encompassing antimicrobial and collagenesis-inducing motifs, to tackle skin and soft tissue infections by exerting a dual effect: antimicrobial protection and faster healing of the wound. This produced peptide 3.1-PP4 showed MIC values as low as 1.0 and 2.1 μM against *Escherichia coli and Pseudomonas aeruginosa*, respectively, and low toxicity to HFF-1 human fibroblasts. Remarkably, the peptide was also potent against multidrug-resistant isolates of *Klebsiella pneumoniae, E. coli, and P. aeruginosa* (MIC values between 0.5 and 4.1 μM), and hampered the formation of/disaggregated *K. pneumoniae* biofilms of resistant clinical isolates. Moreover, this notable hybrid peptide retained the collagenesis-inducing behavior of the reference cosmeceutical peptide C_16_-PP4 (“Matrixyl”). In conclusion, 3.1-PP4 is a highly promising lead toward development of a topical treatment for severely infected skin injuries.

## Introduction

According to the World Health Organization (WHO), antimicrobial resistance is currently disseminated worldwide and can affect anyone, regardless of age, health and socio-economic status (WHO, 2014). Amongst drug-resistant infectious pathogens, which include viruses, parasites, fungi and bacteria, the latter are of special concern in healthcare facilities, as the most severe hospital-acquired infections (HAI) are often associated with multidrug-resistant (MDR) bacteria belonging to the so-called “ESKAPE” group: *Enterococcus faecium, Staphylococcus aureus, Klebsiella pneumoniae, Acinetobacter baumannii, Pseudomonas aeruginosa*, and *Enterobacter* spp. (WHO, 2014). Antibiotic-resistant HAI are life-threatening and greatly increase the risks of standard medical procedures, from major surgery, organ transplantation, or chemotherapy, to management of complicated skin and soft tissue infections (cSSTI). Actually, cSSTI like diabetic foot ulcers (DFU), venous ulcers, pressure ulcers, frequently culminate in hospitalization, where HAI may exacerbate the severity of the infected wounds (Leong et al., 2018). Also, cSSTI may develop as HAI, associated with, e.g., orthopedic or dental implantation (implant-associated infections, IAI) or use of catheters (catheter-associated infections, CAI) (VanEpps and Younger, 2016). Consequently, efficient options for management of cSSTI are urgently needed, especially because their incidence increases with aging. In fact, cSSTI are a mounting burden to both patients and healthcare due to growth of life expectancy: for instance, according to the European Wound Management Association (EWMA, 2014), about 2% of the population in developed countries suffers from chronic wounds, being estimated that 25–50% of hospital beds are occupied by patients with such non-healing injuries, with average costs adding up to 2–4% of the total European budget for healthcare; in the US, the Center for Disease Control estimates that about 50–70% of the 2 million reported HAI are associated to implants or catheters, with mortality rates ranging from <5% for dental implants to >25% for mechanical heart valves (VanEpps and Younger, 2016).

As efficient options to fight MDR bacteria are being exhausted, preclinical and clinical development of antimicrobial peptides (AMPs) has been experiencing a strong impulse (Aminov, 2010; Gomes et al., 2017; Costa et al., 2019). AMPs have broad-spectrum antibacterial activity, and low propensity to induce resistance, hence latest efforts in this area have been focused on the search for AMPs with potent action, particularly against MDR Gram-negative bacteria (Ballantine et al., 2019; Mant et al., 2019; Wang et al., 2019). Findings thereof are quite encouraging toward devising new options to tackle cSSTI like DFU, as the most prevalent bacterial species isolated from these ulcers include, besides the Gram-positive *S. aureus*, several Gram-negative species like *P. aeruginosa*, *E. coli*, *K. pneumonia*, and *Proteus mirabilis* (Ogba et al., 2019). Also, biofilm forming bacteria with a major role in cSSTI, including IAI and CAI, encompass *Staphylococcus epidermidis* (main species associated to IAI and CAI), *S. aureus* (slightly less prevalent than the previous one, but more aggressive), enterococci and Gram-negative bacteria like *E. coli* and *Klebsiella* spp.; in catheter-associated urinary tract infections (UTI), *E. coli* and *Candida* yeasts prevail, whereas Gram-positive pathogens are not so common (VanEpps and Younger, 2016; Li et al., 2019). Still, an efficient treatment of cSSTI should provide not only antibacterial protection, but also promote fast tissue regeneration, which is often deficient in elderly people, especially if bedridden, or affected by diabetes, chronic venous insufficiency, among other conditions (Makrantonaki et al., 2017). Although some AMPs have been reported as having intrinsic skin and soft tissue regenerative properties, in some cases associated with collagenesis-inducing effects (Mangoni et al., 2016; Gomes et al., 2017), most such dual-action AMPs are not potent against Gram-negative bacteria, and/or their activity has not been investigated on MDR clinical isolates and/or bacterial biofilms.

In view of the above, we hypothesized that the conjugation of a collagenesis-inducing, or collagen-boosting peptide (CBP) to an AMP known to be active against both Gram-positive and Gram-negative bacteria might produce dual-action peptide chimeras retaining the properties of their parent CBP and AMP motifs, hence, with potential interest for the management of cSSTI. To this end, the CBP chosen was the matrikine-like peptide KTTKS, also known as “pentapeptide-4” (ahead abbreviated to PP4) whose palmitoylated form (ahead abbreviated to C_16_-PP4) is widely used as a cosmeceutical known as “Matrixyl” (Jones et al., 2013; Choi et al., 2014; Aldag et al., 2016; Schagen, 2017) and reported to possess tissue regenerative properties (Tsai et al., 2007; Park et al., 2017; Krishnamoorthy et al., 2018). As the AMP motif, we chose a previously *de novo* designed synthetic peptide, named 3.1, earlier reported as highly active against both Gram-positive and Gram-negative bacteria (Kang et al., 2009). Both CBP and AMP motifs were conjugated in different ways to produce nine distinct chimeric peptides (Table 1). The *in vitro* activity of these peptide chimeras against relevant bacterial pathogens (both ATCC reference strains and MDR clinical isolates), as well as their toxicity to human fibroblasts (HFF-1), antibiofilm properties, and collagenesis-inducing behavior, were investigated *in vitro*, and revealed a highly promising peptide lead, 3.1-PP4, as next described.

**TABLE 1:** Synthetic CBP/AMP conjugates produced by SPPS.

**Peptide^a^**	**Sequence^b^**	**MW/Da**
3.1	KKLLKWLLKLL	1394.9
C_16_-3.1	Palmitoyl-KKLLKWLLKLL^c^	1633.3
PP4	KTTKS	562.7
C_16_-PP4	Palmitoyl-KTTKS	800.6
PP4-3.1	KTTKSKKLLKWLLKLL	1940.5
C_16_-PP4-3.1	Palmitoyl-KTTKSKKLLKWLLKLL	2179.0
3.1-PP4	KKLLKWLLKLLKTTKS	1940.5
C_16_-3.1-PP4	Palmitoyl -KKLLKWLLKLLKTTKS	2179.0
PP4-βala-3.1	KTTKS-β-Ala-KKLLKWLLKLL^d^	2011.6

**^*a*^All peptides were produced as C-terminal amides. *^*b*^AA residues represented by the single letter code as defined by the IUPAC-IUBMB guidelines on nomenclature and symbolism for amino acids and peptides. ^*c*^Palmitoyl corresponds to the hexadecanoyl group, introduced by coupling hexadecanoic (i.e., palmitic) acid to the peptides’ N-terminus. ^*d*^β-Ala stands for the non-proteinogenic amino acid β-alanine (3-aminopropanoic acid).***

## Materials and Methods

### Peptide Synthesis

Peptides were assembled by solid peptide phase synthesis (SPPS) on an automated Symphony^®^ X synthesizer from Gyros Protein Technologies (Tucson, AZ, United States). The orthogonal Fmoc/tBu scheme was applied (Benoiton, 2006), using a Rink amide MBHA resin (100–200 mesh, 0.52 mmol/g, NovaBiochem) as solid support, which was pre-conditioned in dimethylformamide (DMF, Sigma-Aldrich, St. Louis, MO, United States) for 10 min. The Fmoc protecting group was then removed by treating the resin twice with a solution of 20% piperidine (Sigma-Aldrich, St. Louis, MO, United States) in DMF for 5 min, thus releasing the resin-bound reactive amine groups. The *C*-terminal Fmoc-protected amino acid (Fmoc-AA-OH, Bachem) was next coupled to the deprotected resin, which was treated twice for 10 min with a cocktail solution containing 100 mM of the Fmoc-AA-OH, 100 mM of the *in situ* coupling reagent *O*-(6-chlorobenzotriazol-1-yl)-*N,N,N’,N’*-tetramethyluronium hexafluorophosphate (HCTU, NovaBiochem) and 200 mM *N*-methylmorpholine **(**NMM, Sigma-Aldrich, St. Louis, MO, United States) in DMF. The Fmoc-protecting group was removed as before, to release the amino acid (AA) amine group for subsequent coupling of the next Fmoc-AA-OH. Hence, the peptide chain was grown in the C→N direction through alternating coupling and deprotection cycles, performed as above described, until the full sequence was assembled. For *N*-palmitoylated peptides (as, e.g., C_16_-PP4), after deprotection of the *N*-terminal AA, palmitic acid (Sigma-Aldrich, St. Louis, MO, United States) was coupled by manual synthesis, using an *in situ* coupling cocktail solution containing 5 molar equivalents (eq) of palmitic acid, 5 eq of *N*-ethyl-*N,N*-diisopropylamine (DIEA, Sigma-Aldrich, St. Louis, MO, United States), and 10 eq of *O*-(Benzotriazol-1-yl)-*N,N,N’,N’*-tetramethyluronium hexafluorophosphate (HBTU, NovaBiochem) in DMF. Once fully assembled, the peptides were released from the resins through a 2 h acidolytic cleavage reaction using a cocktail solution containing 95% trifluoroacetic acid (TFA, Sigma-Aldrich, St. Louis, MO, United States), 2.5% triisopropylsilane (TIS, Sigma-Aldrich, St. Louis, MO, United States), and 2.5% of deionized water. The crude peptide thus obtained was purified by a preparative high-performance liquid chromatography (HPLC), on Hitachi-Merck LaPrep Sigma system (VWR) equipped with an LP3104 UV detector and an LP1200 pump, employing a reverse-phase C18 column (250 × 25 mm ID and 5 μm pore size, Merck) and gradient elution using 0.05% TFA in water as solvent A and acetonitrile (ACN, Carlo Erba) as solvent B. The elution method varied according to the specific peptide and all elutions were completed in 60 min, at a 15 mL/min flow-rate. Pure peptide fractions were isolated and pooled, and freeze-dryed to produce the peptide as a low density white solid that was stored at −20°C until further use. Peptide purity was confirmed by analytical HPLC using a Hitachi-Merck LaChrom Elite system equipped with a quaternary pump, a thermostated automated sampler, and a diode array detector; analyses were performed with a reverse-phase C18 column (150 × 4.6 mm ID and 5 μm pore size, Merck) at a 1 mL/min flow rate using a 1–100% of solvent B (ACN) in solvent A, for 30 min, with detection at 220 nm. Peptide structure was confirmed by electrospray ionization-ion trap mass spectrometry (ESI-IT MS).

### Antibacterial Activity

The minimum inhibitory concentration (MIC) of synthetic peptides was determined using the broth microdilution method in cation-adjusted Mueller-Hinton broth (MHB2 – Sigma-Aldrich, St. Louis, MO, United States), according to the recommendations of the Clinical and Laboratory Standards Institute (CLSI, 2012), against four reference strains, namely, *P. aeruginosa* ATCC 27853, *E. coli* ATCC 25922, *S. aureus* ATCC 25923 and *Enterococcus faecalis* ATCC 29212. MIC values of peptide 3.1-PP4 were also determined against MDR clinical isolates of *P. aeruginosa* (PA002, PA004, Pa3, Pa4), *E. coli* (Ec1, Ec2, EC001, EC002, EC003) and *K. pneumoniae* (KP004, KP007, KP010). The peptides were tested in the concentration range of 1–1024 μg/mL. The minimum bactericidal concentration (MBC) was determined as previously reported (Bessa et al., 2018).

### Antibiofilm Activity

The ability of peptide 3.1-PP4 to inhibit the biofilm formation by three *P. aeruginosa* isolates (PA002, PA004, Pa3) and by three *K. pneumoniae* isolates (KP004, KP007 and KP010) was assessed at concentrations equal to the MIC, 1/2 × MIC and 1/4 × MIC in tryptic soy broth – (TSB – Liofilchem s.r.l., Italy) using the crystal violet assay as reported by Gomes et al. (2014). Two independent experiments were performed in triplicate.

The efficacy of peptide 3.1-PP4 on 24 h preformed biofilms of PA002, PA004, KP004, KP007, and KP010 was also evaluated (Gomes et al., 2014). Briefly, biofilms were grown in TSB from a starting inoculum of 1 × 10^6^ CFU/mL in 96-well microtiter plates. After 24 h of incubation at 37°C, the planktonic cells were gently removed and the wells were rinsed and filled with 20 × MIC of the peptide. The optical density at 600 nm (OD_600_) was measured at time 0 and after incubation for 24 h at 37°C. The reduction in the biofilm proliferation was calculated in comparison to the respective non-treated biofilms. Two independent experiments were performed in triplicate.

### Biofilm Metabolic Activity

Twenty four hours biofilms of PA004, KP004, KP007, and KP010 were formed as described above in 96-well microtiter plates and then treated with 20 × MIC of peptide 3.1-PP4 for further 24 h at 37°C. The respective control biofilms were equally formed but in absence of the peptide (only TSB medium was used). Afterward, the bacterial metabolic activity of biofilms was quantified after adding 3-(4,5-dimethylthiazole-2-yl)-2,5-diphenyl tetrazolium bromide (MTT, 0.5 mg/mL – Sigma-Aldrich, St. Louis, MO, United States) for 3 h at 37°C in the dark. DMSO was used to dissolve the formazan crystals formed and the absorbance at 570 nm was measured. Two independent experiments were performed in four replicates.

### Confocal Laser Scanning Microscopy Imaging of Biofilms

For confocal laser scanning microscopy (CLSM) imaging, 24 h biofilms of PA004, KP007, and KP010 were formed in μ-Dish (35 mm, high), ibidi Polymer Coverslips (ibidi GmbH, Planegg-Martinsried, Germany) as previously described by Bessa et al. (2018). Biofilms were non-treated – controls (only medium was used) or treated with 3.1-PP4 at a concentration of 20 **×** MIC. After 24 h, all biofilms were stained using the LIVE/DEAD^TM^ BacLight^TM^ bacterial viability kit (Molecular Probes, Thermo Fisher Scientific, MA, United States). Biofilms were visualized under a laser scanning confocal system Leica TCS SP5 II (Leica Microsystems, Germany), equipped with an inverted (i) microscope Leica DMI6000-CS, using a HC PL APO CS 63x/1.30 Glycerine 21°C objective and the lasers Diode 405 nm and DPSS561 561 nm, and (ii) the LAS AF software.

### Toxicity to Human Fibroblasts

Immortalized human foreskin fibroblasts (HFF-1) were grown as a monolayer from passage number 8–16. For routine maintenance, HFF-1 cells were cultured in Dulbecco’s Modified Eagle Medium (DMEM, from Cell Lines Service) supplemented with 15% fetal bovine serum (FBS, from CLS) and 1% antibiotic/antimycotic solution (100 units/mL of penicillin, 100 μg/mL of streptomycin and 0.25 μg/mL of amphotericin B, from Sigma-Aldrich, St. Louis, MO, United States) at 37°C in an humidified atmosphere with 5% CO_2_. Cells were harvested by trypsinization [0.25% (w/v) trypsin-EDTA4Na, Sigma-Aldrich, St. Louis, MO, United States] twice a week.

The cytotoxicity of synthetic peptides to HFF-1 cells was evaluated using the standard MTT assay. Briefly, cells were seeded at a density of 8 × 10^4^ cells/well onto 96-well plate and incubated at 37°C in a 5% CO_2_ atmosphere. Cells were allowed to grow for 48 h, and serially diluted peptide solutions (0.78–100 μM) were added to the wells. Then, cells were incubated for 72 h at 37°C, after which wells were washed once with phosphate buffered saline (PBS, Sigma-Aldrich, St. Louis, MO, United States), followed by addition of a 0.45 mg/mL MTT solution to each well. Crystals were allowed to form for 1.5 h. Reaction was stopped by rejecting the medium and addition of dimethylsulfoxide (DMSO, Sigma-Aldrich, St. Louis, MO, United States). Absorbance was read at 570 nm (Biotek PowerWave XS).

### Collagenesis-Inducing Effects

Collagen production was assessed as described by Remoué et al. (2013). Briefly, HFF-1 cells were seeded and incubated as described previously for the cytotoxicity assay. After the 72 h incubation with peptides, cells were washed with PBS and the Sirius Red dye in picric acid solution (Sigma-Aldrich, St. Louis, MO, United States) was applied to each well. Cells were incubated at 25°C for 1 h under orbital shaking. Dye was then rejected, and cells were washed twice with absolute ethanol (AGA). Once the wells were dry, 1 M aqueous NaOH (Sigma-Aldrich, St. Louis, MO, United States) was added, and absorbance read at 540 nm (Biotek PowerWave XS).

### Statistical Analysis

The results regarding the biofilm formation, the treatment of preformed biofilms and the biofilm metabolic activity were expressed as mean values ± standard deviation. The statistical significance of differences between controls and experimental groups was evaluated using Student’s *t*-test. *P* < 0.05 were considered statistically significant.

## Results

### Peptide Synthesis

All target peptides (Table 1) were successfully obtained in high purity degrees and presenting ESI-MS data in agreement with the expected molecular weights (MW), as shown in the Supplementary Figures S1–S18.

### Antibacterial Activity and Cytotoxicity

According to CLSI guidelines for antimicrobial susceptibility testing, MIC values were determined for all peptides against ATCC reference strains of two Gram-positive (*S. aureus* ATCC 25923 and *E. faecalis* ATCC 29212), and two Gram-negative (*P. aeruginosa* ATCC 27853 and *E. coli* ATCC 25922) bacterial species, and are shown in Table 2.

**TABLE 2:** MIC and IC_50_ values obtained for the synthetic peptides against four ATCC reference bacterial strains and HFF-1 cells, respectively.

					**IC_50_ ± SEM (μM)^c^**
	**MIC in μg/mL (in μM)**				**on HFF-1 cells**
		
**Peptide**	***E. coli***	***P. aeruginosa***	***S. aureus***	***E. faecalis***	
	****ATCC 25922****	****ATCC 27853****	****ATCC 25923****	****ATCC 29212****	
3.1	8 (6)	4 (3)	4^b^ (3)	4–8 (3–6)	50 ± 3
C_16_-3.1	128 (78.4)	128^b^ (78.4)	>1024 (>627)	256^b^ (158)	23.4 ± 0.7
PP4	>1024 (>1820)	>1024 (>1820)	>1024 (>1820)	>1024 (> 1820)	>100
C_16_-PP4	ND^a^				>100
PP4-3.1	4 (2)	4 (1)	8 (4)	16 (8)	25 ± 2
C_16_-PP4-3.1	32 (15)	64 (29)	64^b^ (29)	64 (29)	4.98 ± 0.08
3.1-PP4	2 (1)	4 (2)	32 (16)	64 (33)	69 ± 5
C_16_-3.1-PP4	64 (29)	64^b^ (29)	128^b^ (59)	64–128^b^ (30–59)	17.8 ± 0.5
PP4-β-ala-3.1	4 (2)	4 (2)	8 (4)	8 (4)	24 ± 2

**^*a*^Not Determined, peptide was insoluble in the medium MHB2. ^*b*^The MBC was 2 × the MIC; in all the other cases, the MBC was equal to the MIC. ^*c*^Data expressed as mean ± SEM of two independent experiments (n = 4–8).**

At the end of the cytotoxicity assays, the number of viable cells was never below that of initially plated cells. As such, data are represented as peptide concentrations causing 50% growth inhibition (IC_50_) on HFF-1 cells, rather than as lethal dosis values. IC_50_ values thus obtained are also included in Table 2.

As expected, the parent CBP, i.e., the matrikine-like peptide PP4, was inactive (MIC > 1024 μg/mL) against all bacterial strains assayed, whereas the parent AMP, peptide 3.1, was highly active against both Gram-positive and Gram-negative bacteria. *N*-palmitoylation of this AMP led to an increase in cytotoxicity and a significant decrease or even loss of antibacterial activity. Relevantly, the hybrid constructs PP4-3.1 and 3.1-PP4, where the parent CBP and AMP motifs were directly linked together in both possible orientations, displayed potent antibacterial activity, which is retained or even improved against Gram-negative bacteria, as compared to that of parent peptide 3.1. Also, while PP4-3.1 was considerably more toxic to HFF-1 cells than parent peptide 3.1 (IC_50_ value of the former is half in comparison to the latter), on the contrary, its reversed analog 3.1-PP4 was less cytotoxic than 3.1. Interestingly, *N*-palmitoylation of any of these conjugates led again to a considerable increase in cytotoxicity and to a decrease in antibacterial activity, whereas insertion of a small flexible linker (β-alanine) between both motifs, as in PP4-β-Ala-3.1 vs. PP4-3.1, did not significantly alter either antibacterial activity or toxicity to HFF-1 cells.

### Activity of Peptide 3.1-PP4 Against MDR Gram-Negative Isolates

In view of the above results, peptide conjugate 3.1-PP4 was selected for further investigation of its activity against MDR isolates of three Gram-negative bacterial species, namely, *P. aeruginosa* (four isolates), *E. coli* (five isolates), and *K. pneumoniae* (four isolates), whose antimicrobial resistance patterns are provided in the Supplementary Table S1. MDR isolates of *K. pneumoniae* were included in this analysis given (i) the high prevalence of *K. pneumoniae* in HAI (Zheng et al., 2017), (ii) that *K. pneumoniae* isolates used are carbanepem-resistant strains of an *Enterobacteriaceae* species, meaning that they are class 1 priority pathogens for the research and development of new antibiotics according to the WHO (WHO, 2017), and (iii) *K. pneumoniae* bacteria are usually better biofilm producers than the other *Enterobacteriaceae* species used, *E.* coli (Reisner et al., 2006). MIC values obtained are shown in Table 3, and confirm peptide’s 3.1-PP4 potent action against Gram-negative bacteria, including MDR isolates, with a selectivity index (SI), i.e., ratio of IC_50_ values for mammalian vs. bacterial cells, ranging from *ca.* 9–69. In fact, for *P. aeruginosa* and *E. coli*, MIC against MDR isolates were in the same range, or below, of those previously found for ATCC reference strains (Table 2). For *K. pneumoniae*, MIC values ranged between 1 and 4 μM, which is remarkable. Moreover, minimal bactericidal concentrations (MBC) were also determined, and found to match the MIC in all cases, which shows that peptide 3.1-PP4 is bactericidal to all MDR isolates tested.

**TABLE 3:** MIC values for 3.1-PP4 against MDR isolates of Gram-negative bacteria.

**Species**	**Isolate**	**MIC in μg/mL (in μM)**
*P. aeruginosa*	PA002	4 (2)
	PA004	2 (1)
	Pa3	2 (1)
	Pa4	2 (1)
*E. coli*	Ec1	4 (2)
	Ec2	2 (1)
	EC001	2 (1)
	EC002	2 (1)
	EC003	1 (0.5)
*K. pneumoniae*	KP010	2 (1)
	KP007	8 (4)
	KP004	4 (2)

### Antibiofilm Activity of Peptide 3.1-PP4

Activity of an antimicrobial agent against planktonic bacteria may not correlate to its action against bacterial biofilms, which are a major cause accounting for the severity and/or chronicity of infections, known as biofilm-associated infections. In fact, the formation of biofilms are described as part of the pathogens’ resistance mechanisms; once established, biofilms represent a stable microbial population that is hidden from the immune system and shielded from antibiotics (Donlan, 2002; Mottola et al., 2016; Omar et al., 2017; Chang, 2018). As such, and since several AMPs have been reported as efficient antibiofilm agents (Yasir et al., 2018; Haney et al., 2019), we have further investigated whether peptide 3.1-PP4 would also display antibiofilm activity. Such activity was assessed in two different stages, (i) in the biofilm formation and (ii) in 24 h mature biofilms. To this end, biofilms of MDR isolates of *P. aeruginosa* and *K. pneumoniae* were used, and results were as described further below. MDR *E. coli* isolates were not good biofilm producers, therefore, they were not used in the antibiofilm activity assays. Indeed, that was not surprising considering the known significantly distinct ability of different *E. coli* isolates to form biofilms *in vitro* (Reisner et al., 2006).

#### Inhibition of Biofilm Formation

**FIGURE 1:**
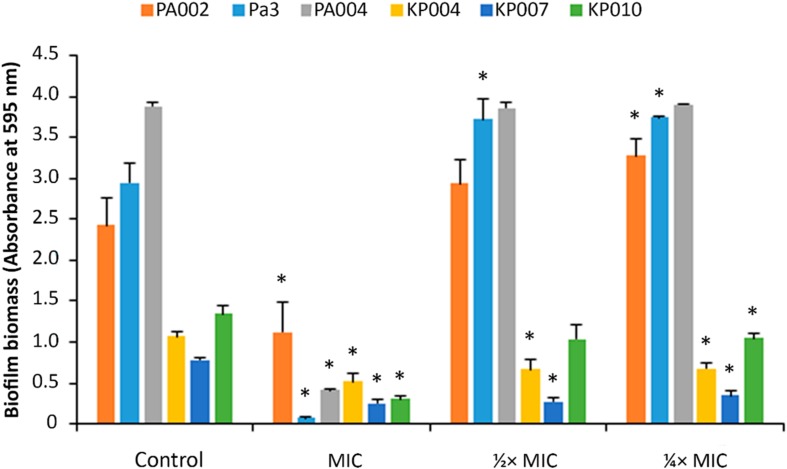
Biofilm biomass quantitation of biofilms formed in the presence of peptide 3.1-PP4. Biofilms of three MDR *P. aeruginosa* (PA002, Pa3, and PA004) and of three MDR *K. pneumoniae* (KP004, KP007, and KP010) were formed in the presence of the peptide at three different concentrations, MIC, 1/2 × MIC, and 1/4 × MIC. Control biofilms were grown in absence of the peptide. Two independent experiments were performed in triplicate. Error bars represent SD. Statistically significant differences between biofilms formed in presence of the peptide and respective control biofilms (*p* < 0.05) are marked with an asterisk (^∗^).

Some of the MDR isolates of *P. aeruginosa* and *K. pneumoniae* were randomly selected to form biofilms in the absence (controls) and presence (at MIC, 1/2 × MIC and 1/4 × MIC) of the peptide 3.1-PP4. In this assay, the test compounds were used at sub-inhibitory concentrations, since if they had been present at the MIC or higher concentrations, bacteria would be killed before starting to produce the biofilms. Hence, by using sub-MIC concentrations, i.e., that are not enough to fully inhibit bacterial growth, it was possible to assess how could the peptide interfere with the normal formation of biofilms. The biofilm biomass formed was next quantitated through the crystal violet assay, and the results are shown in Figure 1. These results are presented as absorbance of crystal violet at 595 nm obtained for each strain in absence (control) and presence of different concentrations (MIC, 1/2 × MIC, and 1/4 × MIC) of the peptide, as this allows for comparison of the different amounts of biofilm formed by each distinct bacterial strain in the control condition.

One immediate observation at Figure 1 shows that *P. aeruginosa* biofilms have significantly higher biomass than those formed by *K. pneumoniae* isolates, indicating that *P. aeruginosa* isolates are stronger biofilm producers in comparison to those of *K. pneumoniae*. An absorbance under 0.5 anticipates that no biofilm was formed (Stepanovic et al., 2007), therefore, as it was expectable, when peptide 3.1-PP4 was present in a concentration equal to the MIC, no biofilm was actually produced by the isolates, with the exception of isolate PA002. Occasionally for some isolates the MIC is different depending on the medium used (Andrews et al., 2002) and indeed the medium used in the biofilm formation, TSB, is different from that used in the MIC determination assay, MHB2. In the case of isolate PA002, the MIC in TSB is higher than in MHB2, and thus the concentration of peptide assayed as being the MIC is in fact a sub-inhibitory concentration. At sub-inhibitory concentrations, i.e., at 1/2 and 1/4 of the MIC, the peptide does not significantly inhibit biofilm formation by any of the three *P. aeruginosa* isolates. However, for *K. pneumoniae* isolates, the peptide is able to reduce the biofilm formed in comparison to the control biofilm.

#### Effects on Preformed Biofilms

Biofilms of MDR *P. aeruginosa* and *K. pneumoniae* isolates were allowed to form for 24 h, then exposed to the peptide at 20-fold its MIC value to ensure that any antibiofilm effects would be visible, since bacterial biofilms are more difficult to eradicate than their planktonic counterparts, and then allowed to grow for another 24 h.

In the case of control biofilms, the second 24 h growth period occurred in the absence of the peptide. Based on the apparent higher efficiency of peptide 3.1-PP4 to inhibit the formation of *K. pneumoniae* than *P. aeruginosa* biofilms, only two MDR isolates of the latter were included in this assay, whereas three MDR *K. pneumoniae* were tested. Optical density of the planktonic phase, as a measure of biofilm proliferation, was reduced in all peptide-treated biofilms, ranging from 3 to 21% reduction on *P. aeruginosa* isolates, and from 34 to 56% reduction on *K. pneumoniae* isolates (Table 4).

**TABLE 4:** Effects of peptide 3.1-PP4, at 20 × MIC, on 24 h preformed MDR bacterial biofilms.

**MDR isolate**	**OD_600_ of the planktonic phase in untreated biofilms**	**OD_600_ of the planktonic phase in peptide-treated biofilms**	**% reduction in biofilm proliferation upon treatment**^a^	****% reduction of metabolic activity in peptide-treated biofilms**^b^**
PA002	0.65 ± 0.03	0.63 ± 0.04	2.8	–
PA004	0.91 ± 0.03	0.71 ± 0.05	21	8.3
KP004	0.63 ± 0.02	0.28 ± 0.00	56	79
KP007	0.56 ± 0.01	0.37 ± 0.03	34	40
KP010	0.64 ± 0.01	0.32 ± 0.02	49	77

**^*a*^Results are the mean of two independent experiments performed in triplicate. ^*b*^Two independent experiments were performed, each in four replicates.**

The influence of the peptide on the metabolic activity of preformed biofilms of one *P. aeruginosa* (PA004) and three *K. pneumoniae* (KP004, KP007, and KP010) isolates was also assessed, through a standard MTT assay. Results, expressed as a percentage of reduction of metabolic activity in peptide-treated vs. untreated biofilms (Table 4), reinforce that peptide 3.1-PP4 significantly affects the viability of MDR *K. pneumonia* isolates, whose metabolic activity can be reduced up to nearly 80% in two of them (KP004 and KP010), and to 40% in the other.

**FIGURE 2:**
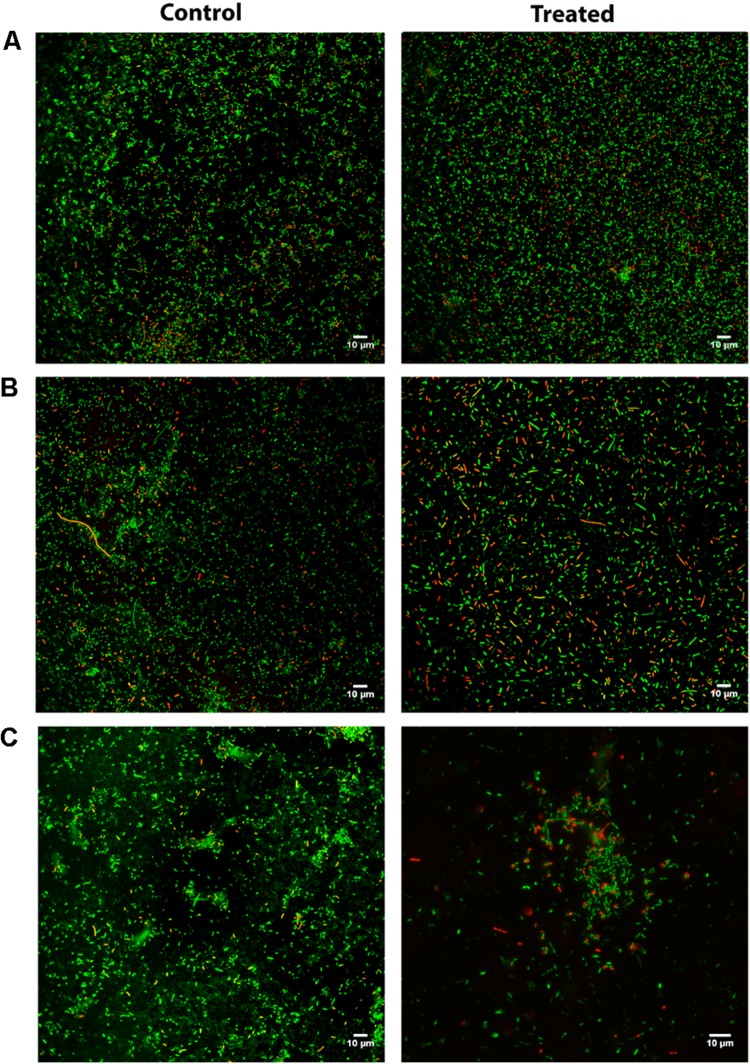
CLSM after Live/Dead staining of *P. aeruginosa* and *K. pneumoniae* biofilms allowed to grow for 24 h and next grown either in absence (control) or presence (treated) of peptide 3.1-PP4 at 20 × MIC; **(A)** PA004 isolate, **(B)** KP007 isolate, and **(C)** KP010 isolate.

To gain further insight into the peptide’s effects on the bacterial biofilms, a Live/Dead staining assay using CLSM was carried out, where untreated and peptide-treated biofilms delivered the images depicted in Figure 2. For this analysis, we selected strains based on their biofilm formation capability, previously assessed by the crystal violet assay (Figure 1 – control condition); hence, we opted to use one strong biofilm producer (*P. aeruginosa* isolate PA004), one good biofilm producer (*K. pneumoniae* isolate KP010), and one weak biofilm producer (*K. pneumoniae* isolate KP007). Noteworthy, only bacterial cells, not biofilm matrix, are stained when the Live/Dead staining is used, which means that only cells within the biofilm are observed. A qualitative analysis of images obtained show that (i) the ratio of red- to green-stained bacterial cells is consistently higher in peptide-treated compared to their respective untreated biofilms, and (ii) 3.1-PP4 has highest impact on the biofilm of isolate KP010, as significant biofilm disaggregation is observed in this case.

### Collagenesis-Inducing Effects

**FIGURE 3:**
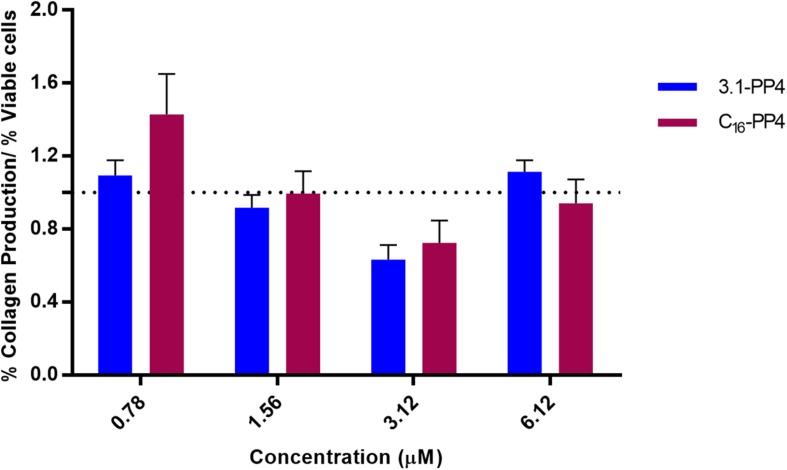
Collagen production by peptide-treated HFF-1 cells, normalized to the number of viable cells for each peptide concentration assayed. No statistically significant differences were observed between the tested peptides at any of the concentrations assayed. The “palmitoyl pentapeptide-4,” C_16_-PP4, also known as Matrixyl, was taken as the reference CBP.

The CBP-AMP conjugates herein addressed include a collagenesis-inducing motif, the amino acid sequence KTTKS whose *N*-palmitoylated derivative is the well-known “Palmitoyl pentapeptide-4” (C_16_-PP4) that emerged in 2000 under the commercial name Matrixyl as an active ingredient in skin rejuvenating cosmetics. Hence, the most promising synthetic peptide 3.1-PP4 was further tested to establish whether or not it exhibited collagenesis-inducing effects comparable to those of C_16_-PP4. To this end, collagen produced by HFF-1 human fibroblasts was determined by the previously validated Picrosirius Red Staining Protocol (Remoué et al., 2013), taking C_16_-PP4 as reference CBP. Results from this type of assay are better interpreted on a comparative basis, as Picrosirius Red staining may occasionally lead to an overestimation of absolute collagen production (Coentro et al., 2017). Results are presented in Figure 3, and expressed as the ratio between collagen produced and the number of viable cells, as the test peptides had different cytotoxicity against the HFF-1 cell line.

The assays were performed at concentrations representative of the MIC values for 3.1-PP4 against MDR isolates of Gram-negative bacteria. Based on the dose-response curves of both compounds, we observed that, within the experimental error, the collagenesis-inducing behavior of peptide 3.1-PP4 was not statistically different from that of Matrixyl (C_16_-PP4). In other words, by replacing the palmitoyl group in Matrixyl with the amino acid sequence of the antimicrobial peptide 3.1, we were able to retain the CBP character of Matrixyl, while converting it into an AMP with potent antibacterial and antibiofilm action on MDR Gram-negative bacteria.

## Discussion

From Fleming’s serendipitous discovery of Penicillin, in 1928, to the end of the 1980’s, many antibiotics have been developed and marketed but, in the last four decades, no truly new antibiotic classes have been disclosed (Fernandes and Martens, 2017; Costa et al., 2019). In the past 4 years, Teixobactin (Ling et al., 2015) and Lungdunin (Zipperer et al., 2016), both cyclic peptides of bacterial origin, were found to have potent action on MDR Gram-positive bacteria. This brought new hope to the management of MDR infections, and refueled the interest on AMP-focused research, which had been cooling down in the first decade of this century. However, Teixobactin, Lungdunin, or synthetic derivatives thereof, are ineffective against Gram-negative bacteria (Ramchuran et al., 2018; Schilling et al., 2019). Hence, seeking for new agents to fight MDR Gram-negative pathogens remains an urgent need and an active field of research.

cSSTI refer to practically all types of severe infections, as soft tissue (SS) includes non-connective tissue like muscles, nerves, and blood vessels, and connective tissue like tendons, ligaments, fascia, nerves, fibrous tissues, fat, and synovial membranes; this leaves out only bone and teeth, the hard tissues in the human body (Skinner, 2006; Stecco et al., 2015). The highly hydrated extracellular matrix (ECM) of SS comprises a gel phase (the so-called ground substance) and a fibrous phase (collagen and elastin), both of which are mainly produced by fibroblasts (Sherman et al., 2015). In view of this, we reasoned that peptide antibiotics effective for the management of cSSTI must combine the properties of an AMP with those of a CBP, which we are pursuing through the synthesis and study of AMP/CBP peptide hybrids like those herein reported.

One first observation regarding these new peptide hybrids was the higher cytotoxicity of the palmitoylated constructs as compared to their non-palmitoylated counterparts. Palmitoylated conjugates were included in this study, due to both the reported superiority of C_16_-PP4 toward PP4 as a collagenesis inducer (Jones et al., 2013; Choi et al., 2014), and literature accounts on the increased bioactivity of AMP upon *N*-acylation with fatty acids (Chicharro et al., 2001; Chu-Kung et al., 2004; Etzerodt et al., 2011; Li et al., 2018). Still, the selectivity of AMP conjugated to fatty acids depends on the size of the latter, with increased toxicity to mammalian cells being often observed for conjugates where fatty acid chains are longer than 14 carbons (Nasompag et al., 2015; Ramachandran et al., 2017), which might explain our results.

The most relevant observation was that all non-palmitoylated peptide conjugates, i.e., PP4-3.1, PP4-β-Ala-3.1, and 3.1-PP4 were markedly active against both Gram-positive and Gram-negative reference bacterial strains, but the latter was the less cytotoxic of the set, being actually safer to human fibroblasts that the reference AMP, peptide 3.1. It was interesting to notice the different behavior of PP4-3.1 as compared to its reversed analog, 3.1-PP4, an observation that comes in line with many reports on the influence of peptide orientation in the antimicrobial properties of peptide-drug conjugates (Aguiar et al., 2019), peptide hybrids (Cavalli et al., 2010), and peptide-grafted materials (Li et al., 2015; Barbosa et al., 2019).

As such, and despite being slightly less potent against Gram-positive bacteria than the other two, peptide 3.1-PP4 was selected for further investigations on its activity against MDR isolates of Gram-negative pathogens in both planktonic and biofilm forms, on which its notable antibacterial efficiency could be confirmed. In addition, the collagenesis-inducing behavior of 3.1-PP4 was not statistically different from that of the reference CBP, peptide C_16_-PP4 (Matrixyl). This is an important observation, as C_16_-PP4 has widely reported superiority toward PP4 alone as a collagenesis-inducer (Jones et al., 2013; Choi et al., 2014), whereas peptide 3.1 has never been reported to have collagen-boosting effects. Notwithstanding, although fully valid for comparative analyses, the collagenesis-inducing activity assay based on Syrius Red may lead to an overestimation of collagen content due (Coentro et al., 2017), hence ongoing work in our lab is targeting quantitation of total collagen deposition by more accurate methods, e.g., enzyme-linked immunosorbent assays (ELISA) (Hosseininia et al., 2016) or quantitative real-time polymerase chain reaction (RT-q-PCR) analysis of procollagen gene expression (Yamazaki et al., 2005). These analyses will include parent unmodified PP4 and 3.1 peptides, as well as their 1:1 mixtures, for a full profiling of collagenesis-inducing activity.

Taking into account its action on *K. pneumoniae* alone, peptide 3.1-PP4 is a novel antimicrobial peptide lead of undeniable relevance: *K*. *pneumoniae* is best known for causing pneumonia, typically as bronchopneumonia or bronchitis, with a quite poor prognosis if harboring antibiotic resistance; but the range of clinical diseases caused by this pathogen is much wider, including UTI, cholecystitis, diarrhea, respiratory tract infections, chronic wound infections, osteomyelitis, meningitis, and sepsis. *K. pneumoniae* can also act as an opportunistic pathogen, especially for people having respiratory dysfunctions such as chronic obstructive pulmonary diseases, among other debilitating conditions. MDR *K. pneumoniae* strains are ubiquitous in healthcare settings, where contact with contaminated medical devices put patients at a serious risk, as sepsis is a real menace once the bacteria enter the bloodstream (Li et al., 2014).

Considering all the above, a wider and deeper study is underway in order to draw a clearer picture on the dual-action potential of new hybrid peptides derived from 3.1 and PP4, and others inspired on them. Thus, additional sets of peptide hybrids are being synthesized that cover (i) peptide *N*-acylation with fatty acids of different lengths, and (ii) modifications aimed at modulating the peptides physico-chemical properties toward improved bioavailability. Moreover, ongoing additional assays on collagenesis-inducing effects are covering a wider range of peptide concentrations, as well as quantitative determination of total collagen deposition levels, where unmodified parent peptides and their 1:1 mixture are also included. Finally, investigation of the antimicrobial/collagenesis-inducing effects of combinations between AMP/CBP hybrid peptides and conventional antibiotics is being pursued, as such mixtures may reveal important synergistic effects (Bessa et al., 2018; Hollmann et al., 2018; Otvos et al., 2018; Zharkova et al., 2019).

## Conclusion

The present disclosure of hybrid peptide constructs combining the wide spectrum antimicrobial peptide 3.1 and the collagenesis-inducing peptide PP4 is unprecedented, and results herein reported demonstrate the potential that such hybrids enclose for the future development of new potent antibacterial agents that are also collagenesis inducers. This is particularly relevant in the context of cSSTI, especially if associated to MDR Gram-negative bacteria. In this work, by replacing the palmitoyl group of the well-known cosmeceutical peptide C_16_-PP4 (Matrixyl) with the antimicrobial peptide 3.1, a new peptide was produced with potent action against Gram-negative bacteria, including MDR isolates of *Enterobacteriaceae*, namely *E. coli* and *K. pneumoniae*. These are the two most prevalent species in UTI of the elderly, and also frequently associated to other cSSTI, and to HAI. The potent action of 3.1-PP4 against *K. pneumoniae* was further confirmed on biofilms of different MDR isolates of this pathogen, whose establishment, growth, and metabolic activity were clearly affected in the presence of the peptide. As such, this work represents a new doorway into the ongoing development of new dual antimicrobial and CBPs inspired on 3.1-PP4, which will be challenged against a wider panel of microbial pathogens, and regarding their full profiling as collagenesis inducers. Results thereof will be timely reported.

## Data Availability

All datasets generated for this study are included in the manuscript and/or the Supplementary Files.

## Author Contributions

AG carried out the peptide synthesis, purification and analysis work, as well as antimicrobial activity assays using reference ATCC strains, and prepared the first version of the full manuscript. LB carried out the antimicrobial and antibiofilm assays using multi-drug resistant clinical isolates. IF carried out the collagenesis-inducing activity assays and peptide cytotoxicity screenings. RF participated in the global scientific reasoning. PGa coordinated the microbiology work. NM coordinated the cytotoxicity and collagenesis-inducing assays. CT participated in the rational design of peptides and experiments, and peptide synthesis work. PGo coordinated the overall study, and participated in the rational design of peptides, and manuscript final revision and submission.

## Conflict of Interest Statement

The authors declare that the research was conducted in the absence of any commercial or financial relationships that could be construed as a potential conflict of interest.
