# Supplementary material for: Turning a Collagenesis-Inducing Peptide Into a Potent Antibacterial and Antibiofilm Agent Against Multidrug-Resistant Gram-Negative Bacteria
Source: Front Microbiol. 2019 Aug 20;10:1915. doi: 10.3389/fmicb.2019.01915 (PMC6710338; doi:10.3389/fmicb.2019.01915)
Supplement: Supplementary file 1 [file Data_Sheet_1.PDF]

Supplementary Material

1 Supplementary Figures and Tables

1.1 Supplementary Table S1

Supplementary Table S1. Antimicrobial resistance pattern of MDR isolates used in this work

**Isolate**	**Antimicrobial resistance pattern**
PA002	AMK, CIP, COL, GEN, TOB
PA004	CIP, GEN, IPM, TOB, TZP
Pa3	ATM, CIP, FEP, GEN
Pa4	ATM, CAZ, CIP, FEP, IPM
Ec1	AMP, ATM, CAZ, CIP, CTX, SXT, TET
Ec2	AMP, ATM, CAZ, CIP, CTX, TET
EC001	AMP, CIP, CXM, SXT, LEV
EC002	AMC, AMP, CIP, CXT, CXM, GEN, LEV, SXT, TOB, TZP
EC003	CIP, CXM, LEV
KP004	AMC, AMP, CAZ, CTX, CXM, ERT, MER, SXT, TZP
KP007	AMC, AMP, CAZ, CIP, CTX, CXM, ERT, IPM, LEV, MER, NIT, SXT, TOB, TZP
KP010	AMC, AMP, CAZ, CIP, CTX, CXM, ERT, IPM, LEV, NIT, TZP

AMC: amoxicillin/clavulanic acid; AMK: amikacin; AMP: ampicillin; ATM: aztreonam; CAZ: ceftazidime; CIP: ciprofloxacin; COL: colistin; CTX: cefotaxime; CXM: cefuroxime sodium; ERT: ertapenem; FEP: cefepime; GEN: gentamicin; IPM: imipenem; LEV: levofloxacin; MER: meropenem; NIT: nitrofurantoin; SXT: Trimethoprim/Sulfamethoxazole; TET: tetracycline; TOB: tobramycin; TZP: piperacillin/Tazobactam

1.2 Supplementary Figures S1 to S18

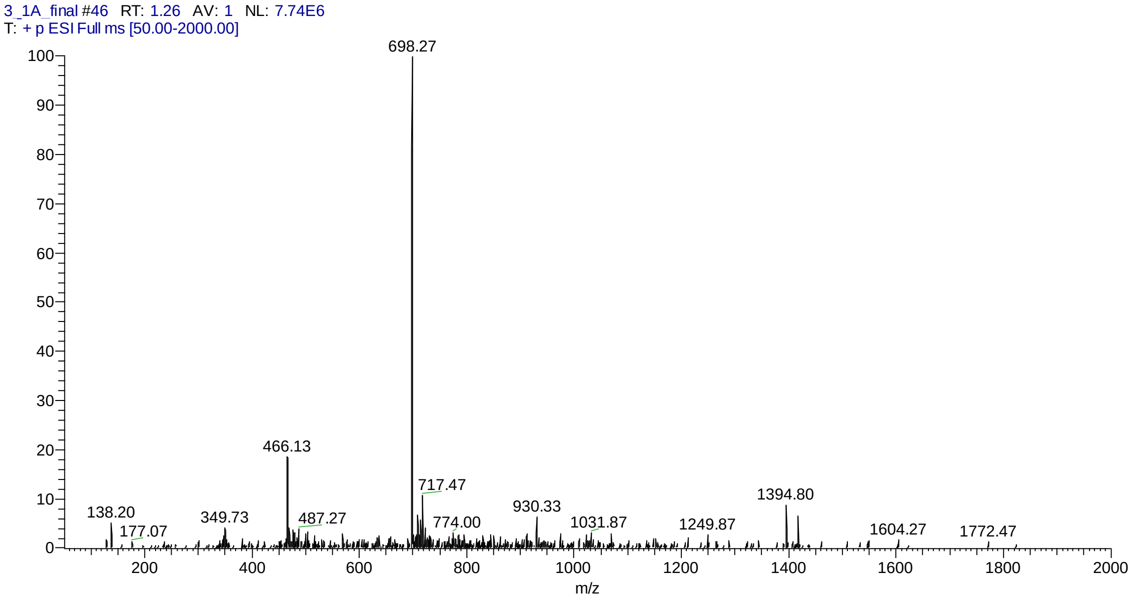

Supplementary Figure S1. ESI-IT mass spectrum (positive mode) of peptide **3.1**

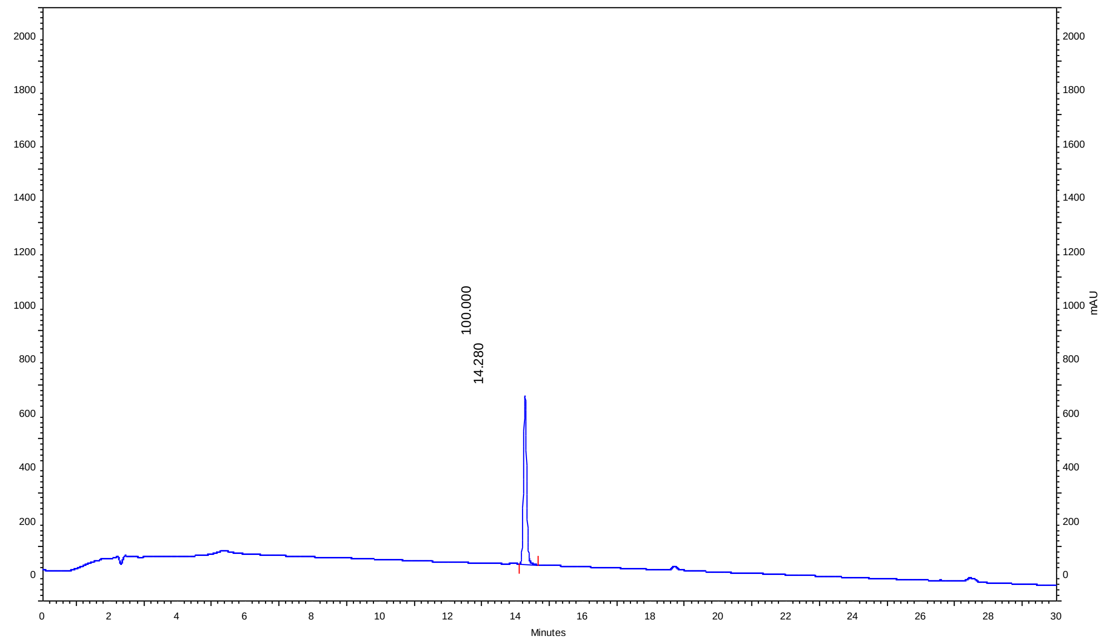

Supplementary Figure S2. HPLC chromatogram of peptide **3.1**

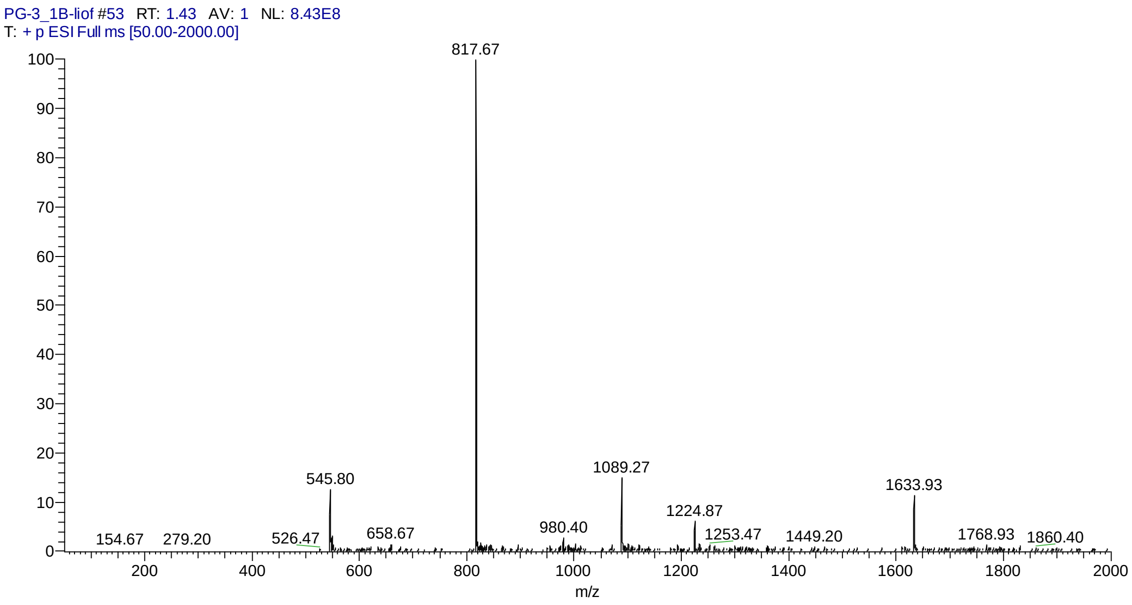

Supplementary Figure S3. ESI-IT mass spectrum (positive mode) of peptide **C_{16}-3.1**

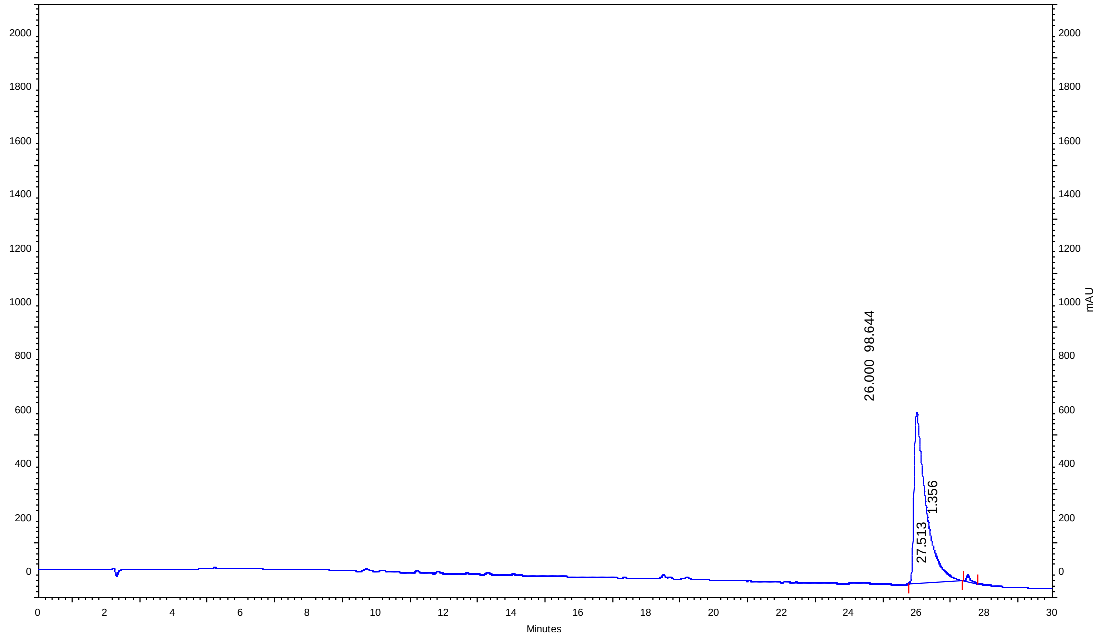

Supplementary Figure S4. HPLC chromatogram of peptide **C_{16}-3.1**

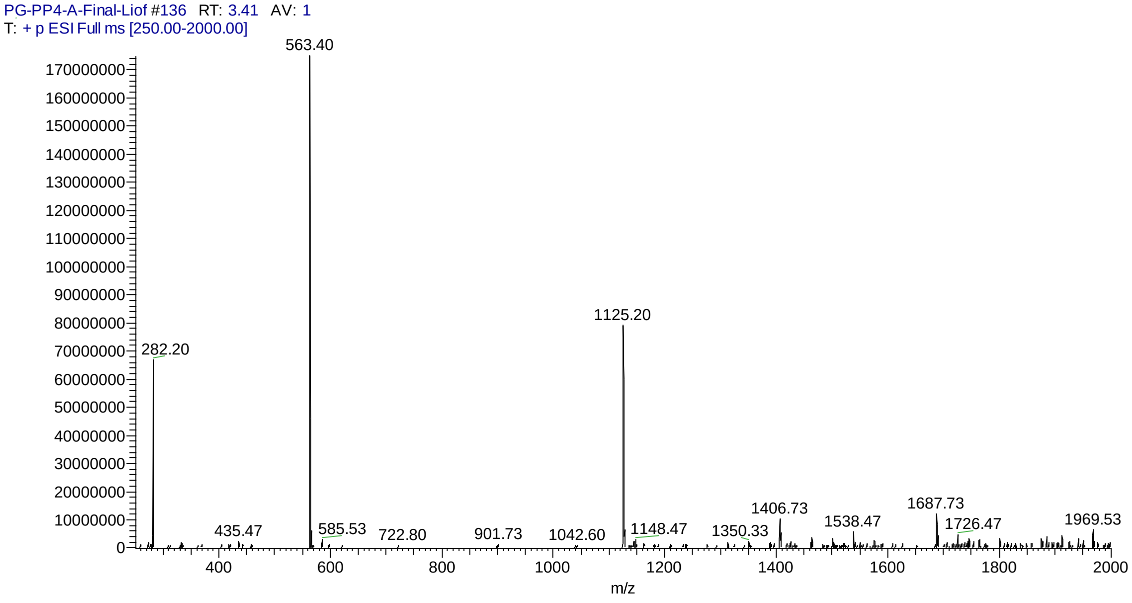

Supplementary Figure S5. ESI-IT mass spectrum (positive mode) of peptide **PP4**

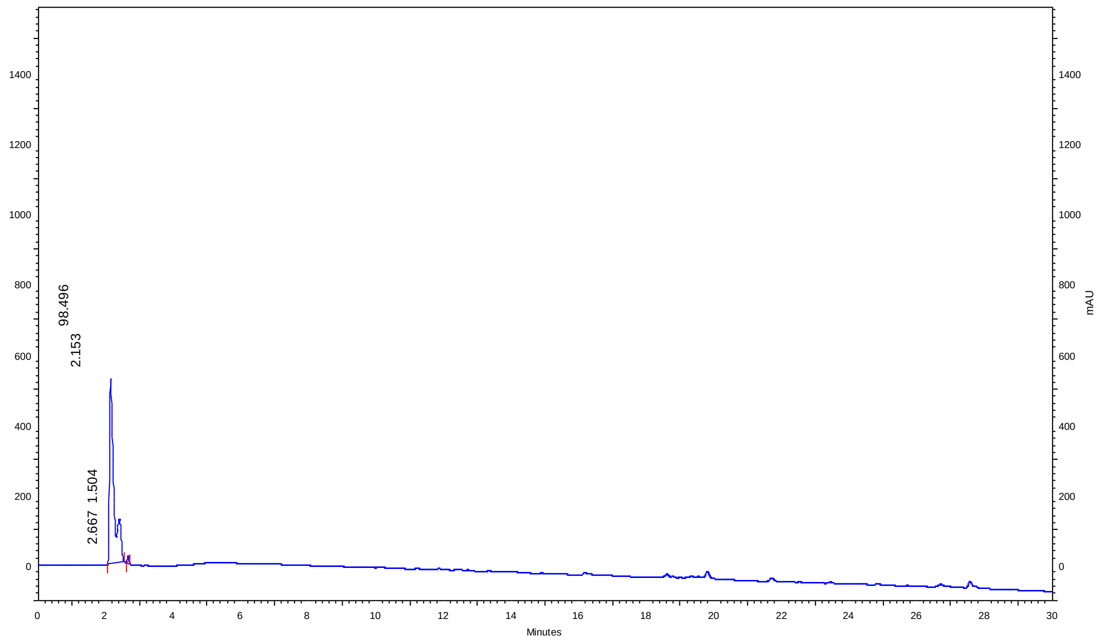

Supplementary Figure S6. HPLC chromatogram of peptide **PP4**

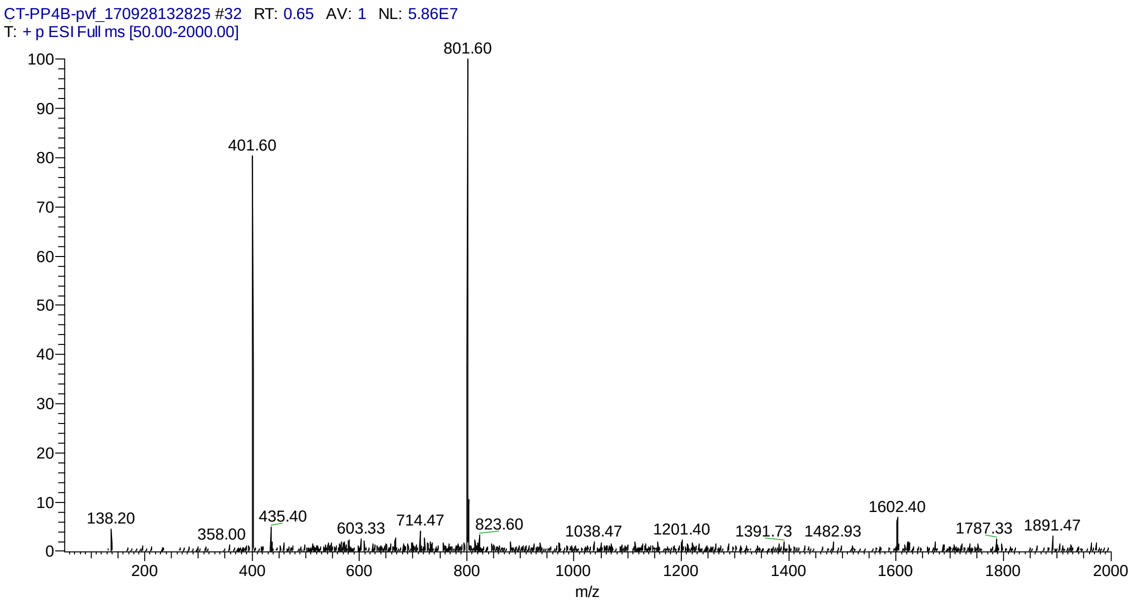

Supplementary Figure S7. ESI-IT mass spectrum (positive mode) of peptide **C_{16}-PP4**

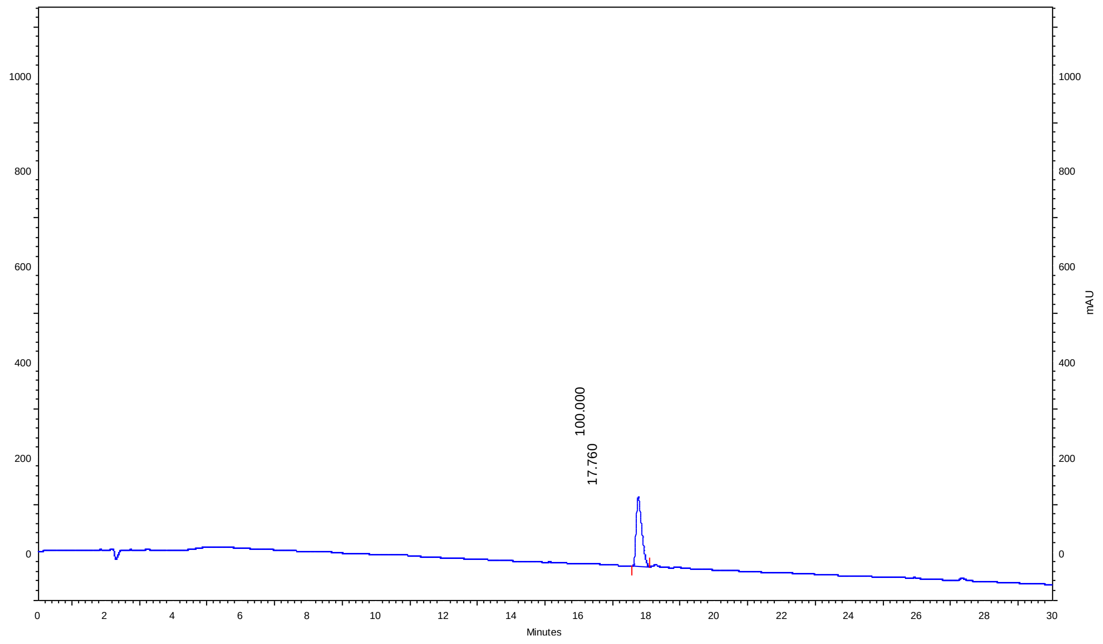

Supplementary Figure S8. HPLC chromatogram of peptide **C_{16}-PP4**

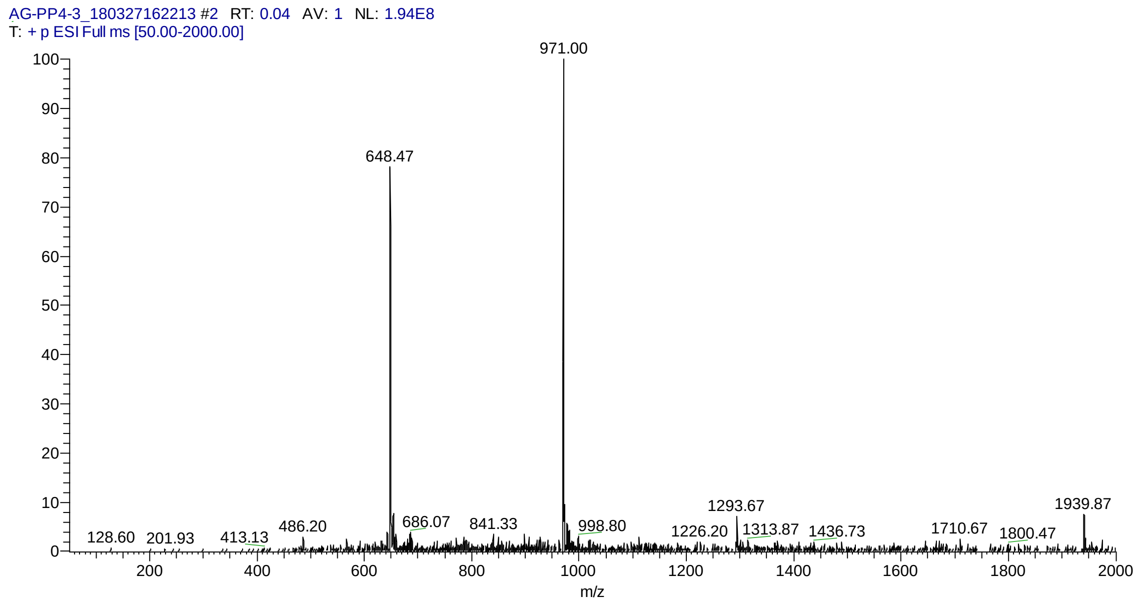

Supplementary Figure S9. ESI-IT mass spectrum (positive mode) of peptide **PP4-3.1**

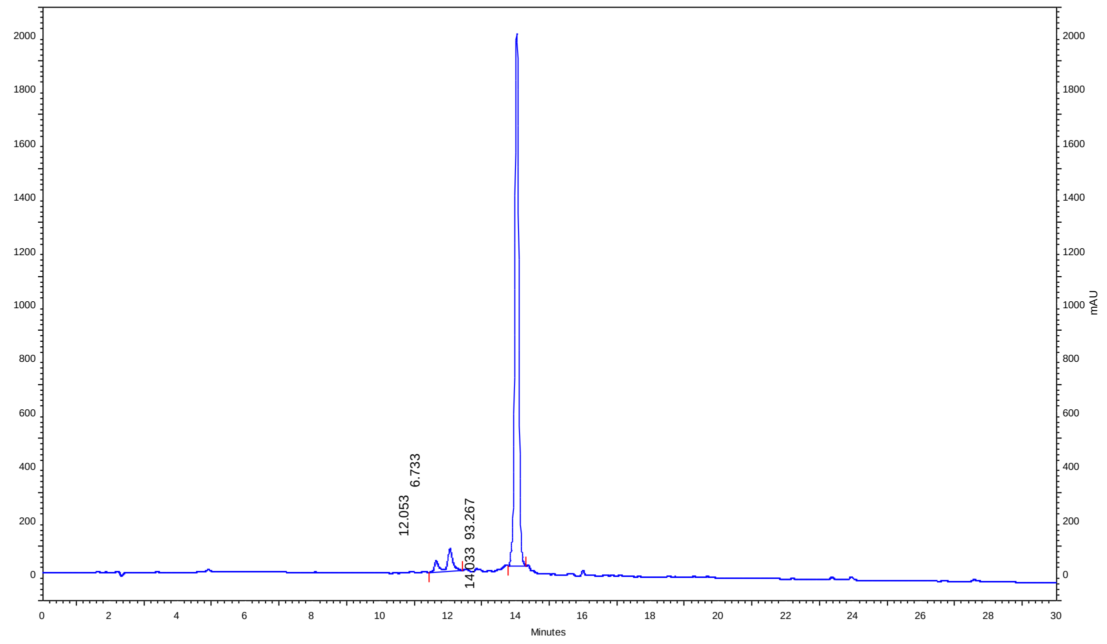

Supplementary Figure S10. HPLC chromatogram of peptide **PP4-3.1**

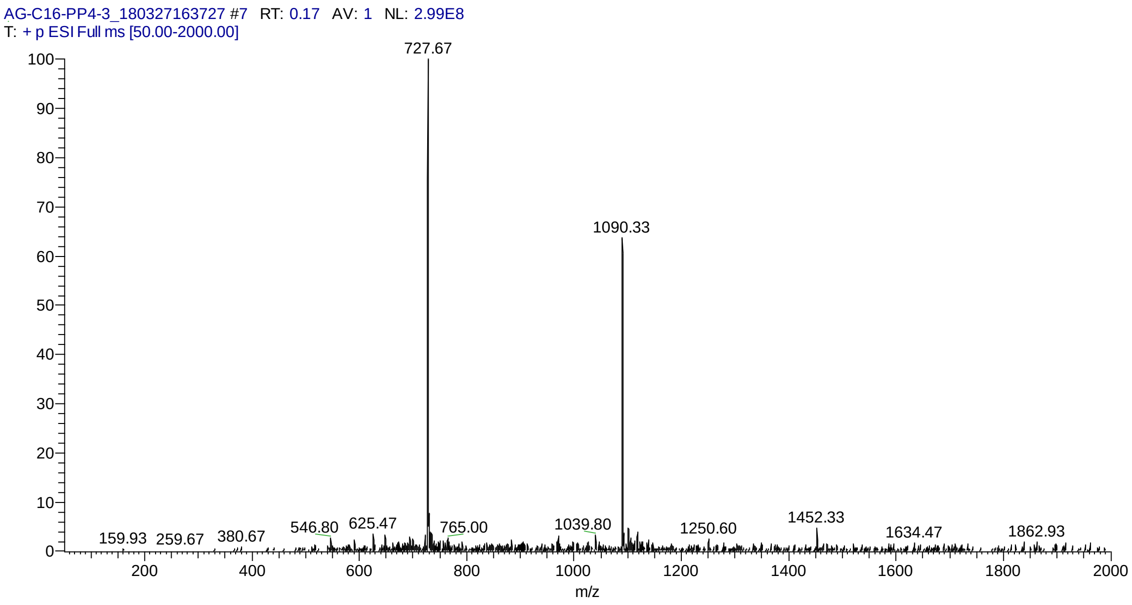

Supplementary Figure S11. ESI-IT mass spectrum (positive mode) of peptide **C16-PP4-3.1**

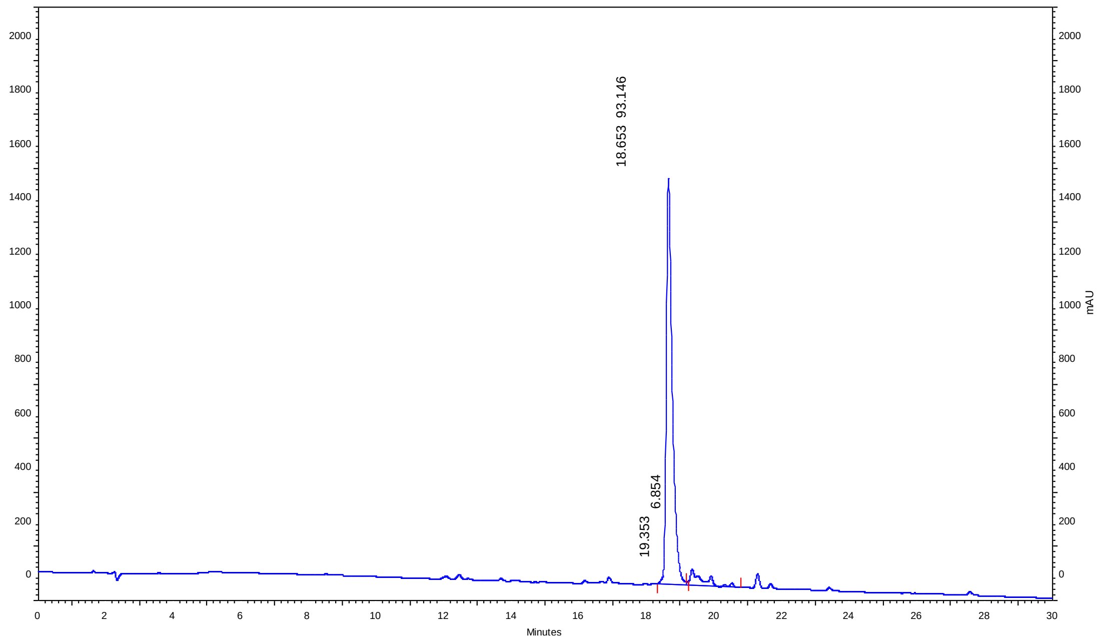

Supplementary Figure S12. HPLC chromatogram of peptide **C16-PP4-3.1**

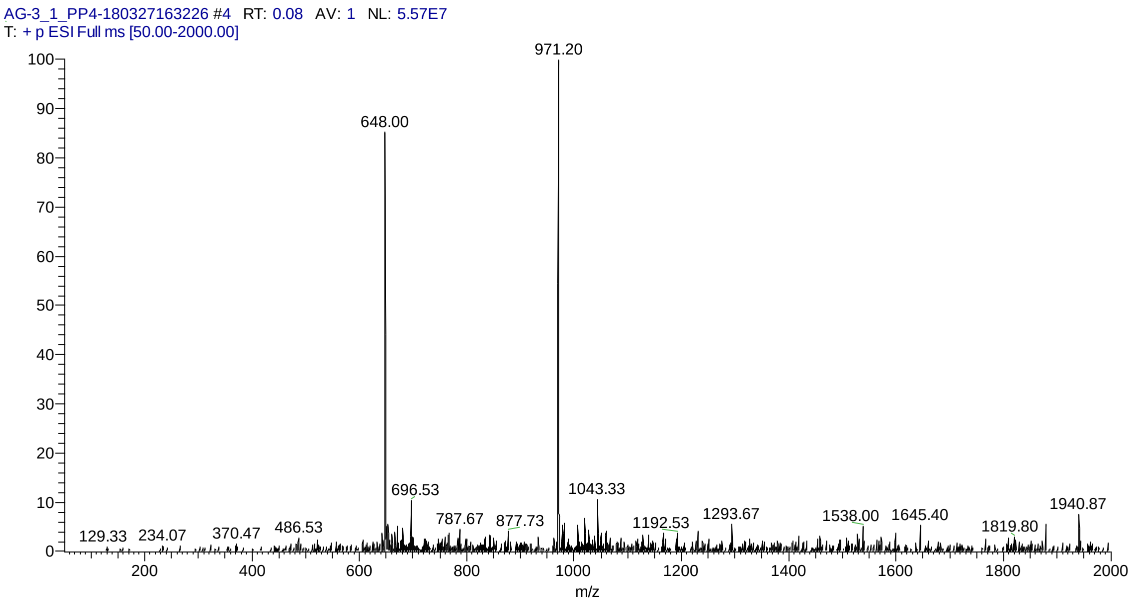

Supplementary Figure S13. ESI-IT mass spectrum (positive mode) of peptide **3.1-PP4**

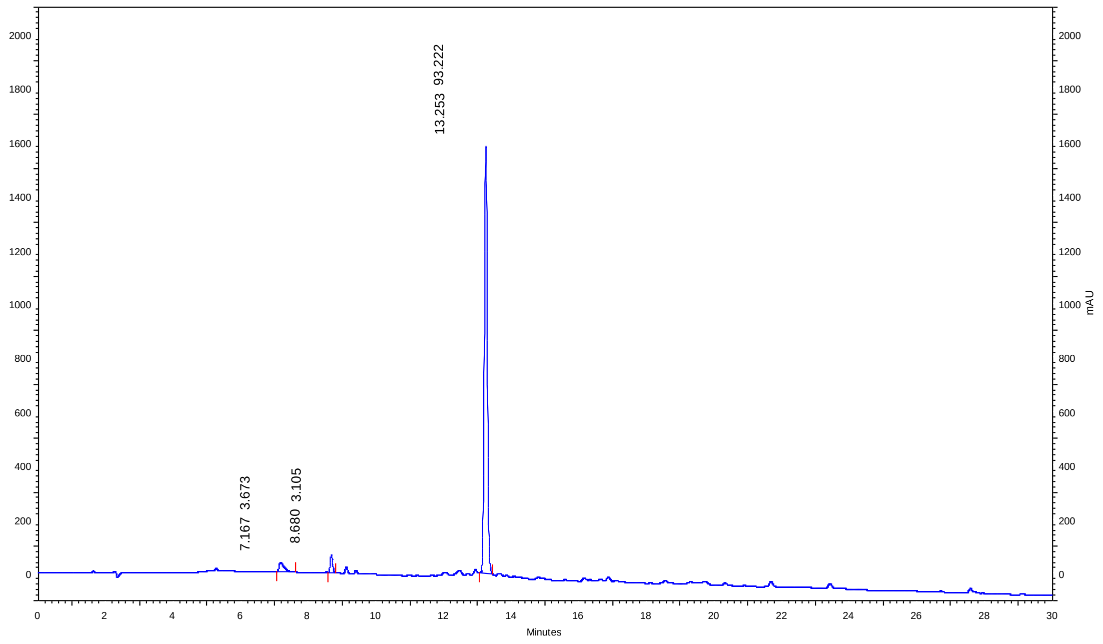

Supplementary Figure S14. HPLC chromatogram of peptide **3.1-PP4**

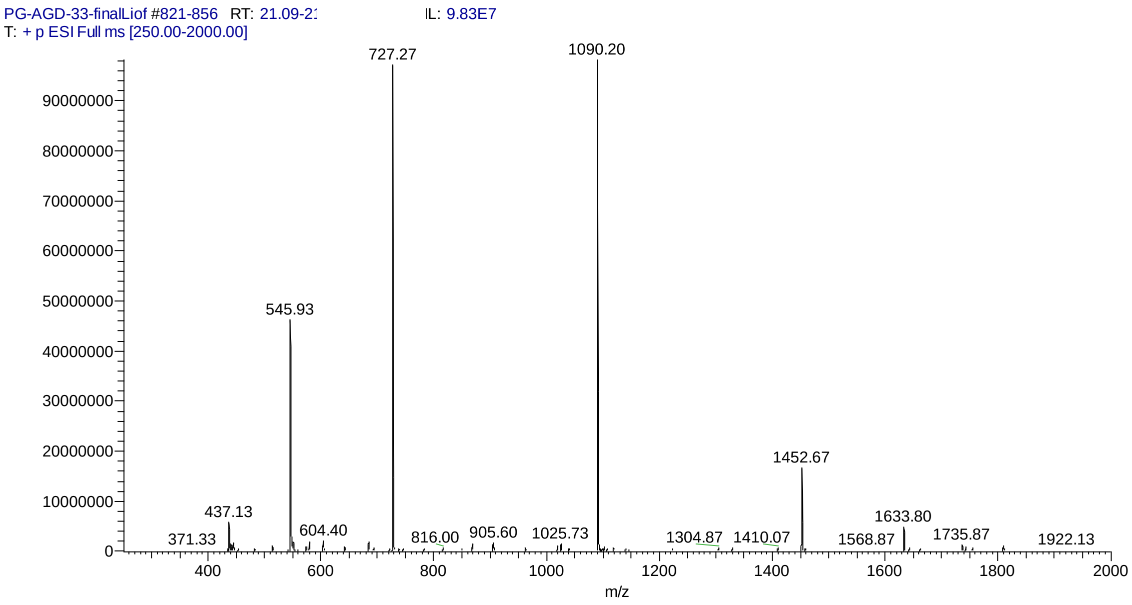

Supplementary Figure S15. ESI-IT mass spectrum (positive mode) of peptide **C16-3.1-PP4**

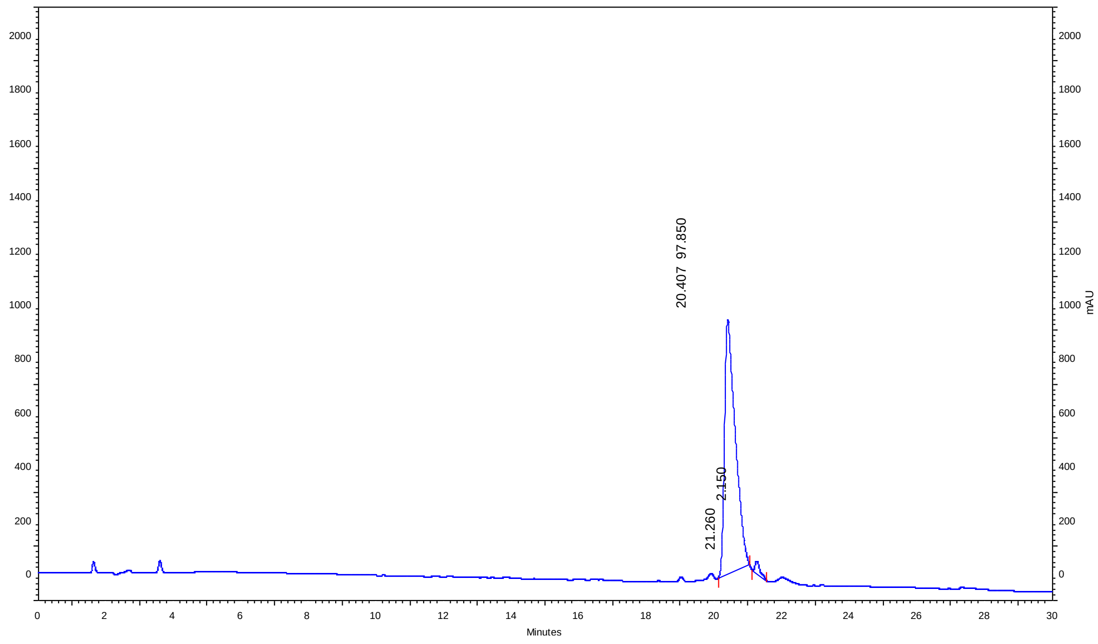

Supplementary Figure S16. HPLC chromatogram of peptide **C16-3.1-PP4**

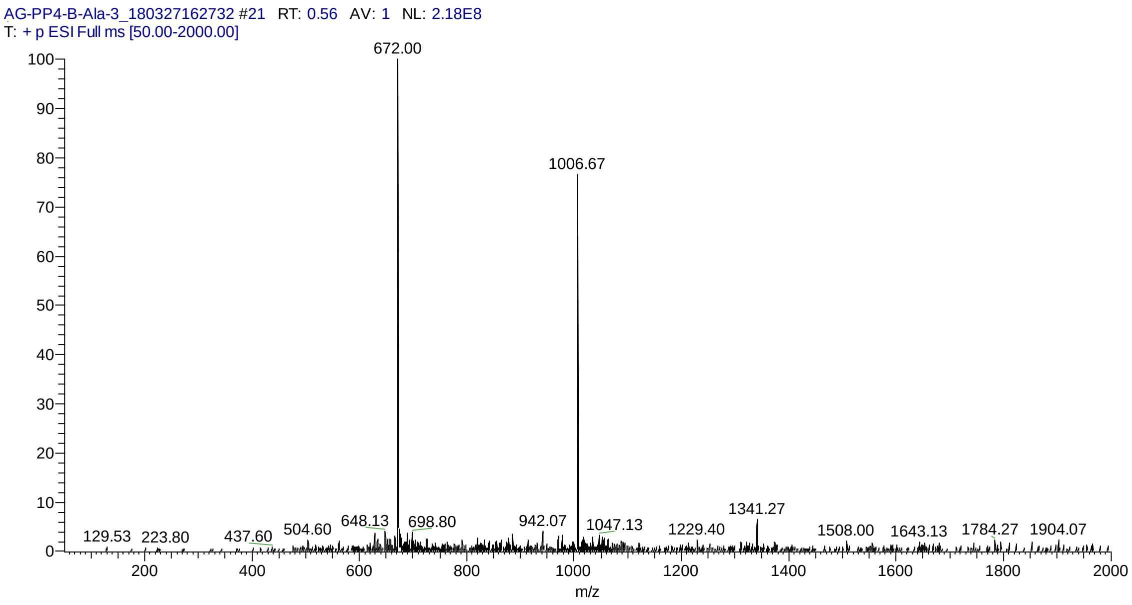

Supplementary Figure S17. ESI-IT mass spectrum (positive mode) of peptide **PP4-βala-3.1**

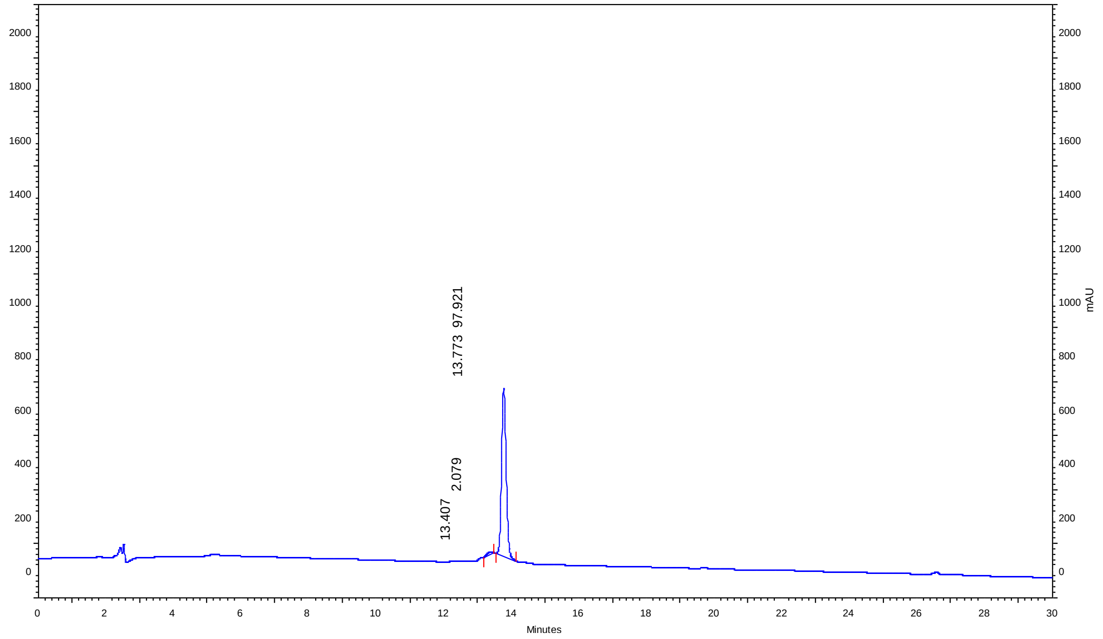

Supplementary Figure S18. HPLC chromatogram of peptide **PP4-βala-3.1**
